# Supplementary material for: Secondary distribution of HIV self-test kits from males to their female sexual partners in two fishing communities in rural Uganda
Source: PLOS Glob Public Health. 2023 Nov 29;3(11):e0002477. doi: 10.1371/journal.pgph.0002477 (PMC10686447; doi:10.1371/journal.pgph.0002477)
Supplement: Attachment — Submitted filename: PGPH-D-23-01082_reviewer.pdf [file pgph.0002477.s003.pdf]

PLOS Global Public Health

Secondary distribution of HIV self-test kits from male fisherfolk to their female sexual partners in two fishing communities in rural Uganda

--Manuscript Draft--

**Manuscript Number:**	PGPH-D-23-01082
**Article Type:**	Research Article
**Full Title:**	Secondary distribution of HIV self-test kits from male fisherfolk to their female sexual partners in two fishing communities in rural Uganda
**Short Title:**	Secondary HIV self-test kits distribution from males to females
**Corresponding Author:**	Joseph KB Matovu, Ph.D.<br>Makerere University School of Public Health<br>Kampala, UGANDA
**Order of Authors:**	Joseph KB Matovu, Ph.D.
	Linda Kemigisha
	Geoffrey Taasi
	Joshua Musinguzi
	Rhoda K. Wanyenze
	David Serwadda
**Keywords:**	Secondary distribution; HIV self-test kits; male fisherfolk; female sexual partners; fishing communities; Uganda
**Abstract:**	Introduction<br><br>Secondary distribution of HIV self-test kits from females to their male partners has increased HIV testing rates in men in most settings but little evidence exists on the potential for secondary distribution of HIV self-test kits from males to their female partners. We assessed the acceptability of secondary HIV self-test kits distribution from male fisherfolk to their female sexual partners in a fishing community context.<br><br>Methods<br><br>This secondary analysis used data from the PEer-led HIV Self-Testing intervention forMEN (PEST4MEN), an ongoing pilot interventional study in Buvuma and Kalangala island districts within the Lake Victoria basin in Uganda. At the baseline visit in July 2022, data were collected from 400 male fisherfolk aged 15+ years who self-reported a HIV-negative or unknown HIV status. Enrolled men were asked to pick two oral fluid HIV self-test kits from a trained male distributor. At the first follow-up visit, in September 2022, men were asked about the number of kits that they received; if they gave out kits to anyone, and, if they did, whether or not the recipients used them to self-test for HIV. Data were analyzed using STATA version 16.0.<br><br>Results<br><br>Of 361 men interviewed at follow-up, 98.3% (355) received at least one kit; 79.7% (283) received two kits. Of the men that received two kits, 88.0% (181) gave the second kit to someone else; 97.8% (n=177) accepted to take the kit. Seventy-five per cent of the men (132/177) reported that they gave the second kit to a female sexual partner; 87.1% (n=115) of the female sexual partners used the kits to self-test for HIV. Ninety-seven per cent (n=112) of the men reported that they knew their sexual partners' HIV self-test results. Of these, 93.7% (n=105) reported that their partners were HIV-negative while 6.3% (n=7) reported that they were HIV-positive. Of the HIV-positive female partners, only 28.6% (n=2) were linked to HIV care.<br><br>Conclusion

Powered by Editorial Manager® and ProduXion Manager® from Aries Systems Corporation

	Secondary distribution of HIV self-test kits from male fisherfolk to their female sexual partners is well accepted by women in the fishing communities. However, innovative efforts are needed to link newly diagnosed HIV-positive women to HIV care.
**Additional Information:**	
**Question**	**Response**
**Financial Disclosure**<br><br>Enter a financial disclosure statement that describes the sources of funding for the work included in this submission and the role the funder(s) played. This includes grants and any commercial funding of the work or authors.<br><br>This statement will be typeset if the manuscript is accepted for publication.<br><br>*Please review the submission guidelines and the instructions link below for detailed requirements and guidance.*	This study is part of the EDCTP2 programme that is supported by the European Union (under grant number TMA2019CDF-2729-PEST4MEN, PI: Joseph Matovu). The funders had no role in the study design, data collection and analysis, decision to publish, or preparation of the manuscript.
**Competing Interests**<br><br>On behalf of all authors, disclose any competing interests that could be perceived to bias this work.<br><br>This statement will be typeset if the manuscript is accepted for publication.<br><br>*Please review the instructions link below and PLOS Global Public Health's competing interests policy to determine what information must be disclosed at submission.*	The authors declare that they have no competing interests
**Data Availability**<br><br>Before publication, Authors are required to make fully available and without restriction all data underlying their findings. Please see our PLOS Data Policy page for detailed information on this policy.	The dataset used for the analysis has been supplied as supplementary file 1.

A **Data Availability Statement**, detailing where the data can be accessed, is required at first submission. Insert your Data Availability Statement in the box below.<br><br>Please see the data reporting section of our submission guidelines for instructions on what you need to include in your Data Availability Statement.<br><br>This statement will be typeset if the manuscript is accepted for publication.<br><br>*PLOS allows rare exemptions to address legal and ethical concerns. If you have legal or ethical restrictions, please use the box below to detail these in full sentences for the Journal team to consider.*	

Powered by Editorial Manager® and ProduXion Manager® from Aries Systems Corporation

Manuscript

Click here to access/download;Manuscript;Secondary_HIVST_kits_distribution

Secondary distribution of HIV self-test kits from male fisherfolk to their female sexual partners in two fishing communities in rural Uganda

Joseph KB Matovu[1,2], Linda Kemigisha[2], Geoffrey Taasi[3], Joshua Musinguzi[3], Rhoda K. Wanyenze[2], David Serwadda[2]

[1]Busitema University Faculty of Health Sciences, Mbale, Uganda
[2]Makerere University School of Public Health, Kampala, Uganda
[3]Ministry of Health, Kampala, Uganda

Short title: Secondary HIV self-test kits distribution from males to females

***Correspondence author**
Email: jmatovu@musph.ac.ug (JKBM)

Abstract

Introduction: Secondary distribution of HIV self-test kits from females to their male partners has increased HIV testing rates in men in most settings but little evidence exists on the potential for secondary distribution of HIV self-test kits from males to their female partners. We assessed the acceptability of secondary HIV self-test kits distribution from male fisherfolk to their female sexual partners in a fishing community context.

Methods: This secondary analysis used data from the **PE**er-led HIV **S**elf-**T**esting intervention **for MEN** (PEST4MEN), an ongoing pilot interventional study in Buvuma and Kalangala island districts within the Lake Victoria basin in Uganda. At the baseline visit in July 2022, data were collected from 400 male fisherfolk aged 15+ years who self-reported a HIV-negative or unknown HIV status. Enrolled men were asked to pick two oral fluid HIV self-test kits from a trained male distributor. At the first follow-up visit, in September 2022, men were asked about the number of kits that they received; if they gave out kits to anyone, and, if they did, whether or not the recipients used them to self-test for HIV. Data were analyzed using STATA version 16.0.

Results: Of 361 men interviewed at follow-up, 98.3% (355) received at least one kit; 79.7% (283) received two kits. Of the men that received two kits, 88.0% (181) gave the second kit to someone else; 97.8% (n=177) accepted to take the kit. Seventy-five per cent of the men (132/177) reported that they gave the second kit to a female sexual partner; 87.1% (n=115) of the female sexual partners used the kits to self-test for HIV. Ninety-seven per cent (n=112) of the men reported that they knew their sexual partners' HIV self-test results. Of these, 93.7% (n=105) reported that their partners were HIV-negative while 6.3% (n=7) reported that they were HIV-positive. Of the HIV-positive female partners, only 28.6% (n=2) were linked to HIV care.

Conclusion: Secondary distribution of HIV self-test kits from male fisherfolk to their female sexual partners is well accepted by women in the fishing communities. However, innovative efforts are needed to link newly diagnosed HIV-positive women to HIV care.

Key words: Secondary distribution, HIV self-test kits, male fisherfolk, female sexual partners, fishing communities, Uganda

Introduction

Secondary distribution of HIV self-test kits, a process through which HIV self-test kits are delivered to potential users through someone else, has been used to increase HIV testing uptake among men and other unreachable populations (e.g. men who have sex with men, female sex workers) in most settings [1]. In sub-Saharan Africa, the most common form of secondary HIV self-test kits distribution has been from women to their male partners [2-4]. For instance, evidence shows that pregnant women can successfully deliver HIV self-test kits to their male partners [5-9], and that women can use a variety of strategies to achieve this purpose [10-12]. Studies show that secondary HIV self-test kits distribution from pregnant women to their male partners almost doubled HIV testing uptake among men in Uganda and Kenya [5-8]. There is also growing evidence that social network-based secondary distribution of HIV self-test kits from men to fellow men is well accepted among men in the general population [13, 14] as well as among key populations, including men who have sex with men [15-17]. These findings indicate that secondary distribution is an acceptable approach for reaching men and other unreachable populations with HIV self-testing services.

However, while there is growing evidence of the acceptability of secondary distribution of HIV self-test kits from women to their male partners, there are virtually no studies that have assessed the secondary distribution of HIV self-test kits from men to their female partners. In societies where men make key decisions in the relationship, including decisions on health matters, such as in sub-Saharan Africa [18, 19], understanding whether or not men would accept to take HIV self-test kits to their female partners would be essential in addressing gender norms that normally affect women's decision-making autonomy regarding when, where and how to access health services [20,

21]. Besides, since men tend to fear to test for HIV with their female partners [22], encouraging them to take HIV self-test kits to their female partners could encourage them to test with them, thereby promoting couples' HIV testing uptake in steady relationships. However, it is possible that women may fear to accept HIV self-test kits delivered to them by their male partners for fear of being blamed for bringing HIV into the relationship in the event that they (the female sexual partners) turn out to be HIV-positive [23, 24]. Thus, studies are urgently needed to assess if women would accept to receive and use HIV self-test kits delivered by their male sexual partners.

Studies show that women in the fishing communities tend to be highly mobile [25, 26], moving from one place to the other due to work-related purposes. Because of their high mobility, women may not only be missed through conventional HIV services but they may also have no time to attend facility-based HIV testing services. Such women would benefit from HIV self-testing services when HIV self-test kits have been delivered to them by their male partners. Our study aimed to assess the acceptability of secondary distribution of HIV self-test kits from male fisherfolk to their female sexual partners in two high HIV prevalence island districts in rural Uganda.

Materials and Methods

Study design, site and population

This secondary analysis used data collected as part of an ongoing, pilot HIV self-testing interventional study that is being implemented in two fishing communities (one in each district) in two high HIV prevalence island districts (Buvuma and Kalangala) located in the Lake Victoria

basin in Uganda. Estimates put HIV prevalence in Buvuma at 14% [27] and in Kalangala at 18.8% [28], much higher than the national average of 5.5% among adults aged 15-49 years [29]. The primary objective of the pilot study was to improve HIV testing uptake and linkage to HIV care among male fisherfolk through a peer-led HIV self-testing intervention for men (PEST4MEN). The PEST4MEN study methodology has been described previously (Matovu et al. *in press*). In brief, the intervention aimed to improve HIV testing among male fisherfolk through giving HIV self-test kits to trained male distributors in existing social networks (hereafter referred to as "peer-leaders") to distribute to fellow men within their own social networks. Prior to enrolling in the study, men had to have been nominated and seconded to the study by a trained peer-leader from a documented social network, and all nominated men had to be screened for study eligibility prior to the baseline interview. Eligible men had to self-report a HIV-negative result or unknown HIV status and should not have tested for HIV at least three months from the time of enrolment. Eligible men were administered a baseline questionnaire and asked to go to the peer-leader who nominated them to pick two oral fluid HIV self-test kits, one for themselves and the other for someone else, including their female sexual partners.

Data collection procedures and methods

Data for the pilot study were collected from 400 men at baseline (July 2022) and 361 at the first follow-up visit (September 2022). Baseline data were collected on socio-demographic and behavioral characteristics, including HIV testing history and sexual behaviors and willingness to use HIV self-test kits if they were availed to them free of charge, among other characteristics. At the first follow-up visit, men were asked whether or not they had given out the second kit to anyone else, and if they did, we asked them about who they gave the second kit to, whether or not that

person accepted to take the kit, and whether or not they think that the person they gave the second kit to actually used it to self-test for HIV. Men who reported that the person that they gave the kit to used it to self-test for HIV were asked if they knew that person's HIV status, and if HIV-positive, if they knew whether or not that person linked to HIV care. No attempt was made to interview the female partners who received the second kit; data were collected from the male fisherfolk enrolled into the study. Data were collected by a team of six Research Assistants using questionnaires configured on KoboCollect-enabled mobile phones.

Measures

The primary outcome was acceptability of HIV self-test kits by female sexual partners, defined as female sexual partners accepting to take kits given to them by their male partners and use them to self-test for HIV. The secondary outcome was linkage to HIV care among female sexual partners who were reported to have received an HIV-positive self-test result.

Data analysis

We conducted descriptive statistics to compute the percentage of men that received two HIV self-test kits and, of these, the percentage of men who gave out the second kit to anyone else. Of those that gave out the kits to anyone else, we determined the proportion of men who reported that they gave the second kit to their female sexual partners. We then determined the proportion of men that reported if the female sexual partner accepted to take the kit, and what percentage of men reported that their female sexual partners used the kits to self-test for HIV. Among men who reported that their female sexual partners self-tested for HIV, we determined the proportion of those who reported that their female sexual partner self-reported their HIV self-test results to them. Of those

that reported an HIV-positive female sexual partner, we asked if the female partner had linked to HIV care. Comparisons between proportions were made using Pearson’s Chi-square tests. Data analysis was conducted using STATA statistical software, version 16.0.

Ethical considerations

This study was conducted according to the principles expressed in the Declaration of Helsinki. The protocol was approved by the School of Public Health Research and Ethics Committee (Protocol Number: SPH-2021-158) and cleared by the Uganda National Council for Science and Technology (HS2034ES), as per national research guidelines for research among human subjects. All participants provided written informed consent prior to participating in the interviews.

Results

Table 1 shows the characteristics of the 400 male fisherfolk that were interviewed at baseline. Of these, 52.7% (n=211) were from Buvuma while 47.2% (n=189) were from Kalangala district. Overall, 68.0% (n=272) were aged 15-34 years, 64.2% (n=257) had primary education as their highest level of education, while 56.7% (n=227) were engaged in fishing or fishing-related activities. Nearly 60% (n=233) of the men were currently married, with a higher proportion of men in Buvuma reporting that they were currently married than those in Kalangala district (69.2%, n=146 *vs.* 46.0%, n=87, $P<0.0001$). Considering those who were currently married and other men who were in a sexual relationship but not married, up to 83.7% (n=335) can be said to have been in a sexual relationship with a female sexual partner. There were more men in Buvuma who were

engaged in fishing or fishing-related activities than in Kalangala district (61.1% [n=129] *vs.* 51.8% [n=98], *P*=0.06). Eighty-two per cent (n=328) of the men had ever tested for HIV; higher in Kalangala (88.9%, n=168) than in Buvuma (75.8%, n=160).

Table 1: Baseline socio-demographic characteristics of male fisherfolk in the fishing communities, overall and by study district

Characteristic	Kalangala (N=189, n(%)])	Buvuma (N=211, n(%)])	Total (N=400, n(%)])
**Age-group**			
15-24 years	59 (31.2)	66 (31.3)	125 (31.2)
25-34 years	68 (36.0)	79 (37.4)	147 (36.7)
35-44 years	44 (23.3)	50 (23.7)	94 (23.5)
45+ years	18 (9.5)	16 (7.6)	34 (8.5)
**Highest level of education attained**			
No education	10 (5.3)	19 (9.0)	29 (7.2)
Primary education	125 (66.1)	132 (62.6)	257 (64.2)
Post-primary	54 (28.6)	60 (28.4)	114 (28.5)
**Marital status**			
Never married/not in any relationship	13 (6.9)	13 (6.2)	26 (6.5)
Never married but in a relationship	44 (23.3)	37 (17.5)	81 (20.3)
Currently married	87 (46.0)	146 (69.2)	233 (58.2)
Ever married, not in a relationship	28 (14.8)	11 (5.2)	39 (9.7)
Ever married, in a relationship	17 (9.0)	4 (1.9)	21 (5.2)
**Occupation**			
Fishing	65 (34.4)	88 (41.7)	153 (38.2)
Fishing-related activity	33 (17.5)	41 (19.4)	74 (18.5)
Business/commercial	39 (20.6)	34 (16.1)	73 (18.2)
Other occupation	52 (27.5)	48 (22.7)	100 (25.0)
**Ever tested for HIV**			
Yes	168 (88.9)	160 (75.8)	328 (82.2)
No	21 (11.1)	51 (24.2)	71 (17.8)

Table 2 shows the number and proportion of men that received HIV self-test kits from their peer-leaders in Kalangala and Buvuma districts, as interviewed at the first follow-up visit. Of the 400 men interviewed at baseline, 90.2% (n=361) were interviewed at the first follow-up visit. Of these, 98.3% (n=355) reported that they received HIV self-test kits from their peer-leaders. Nearly all men

(99.4%, n=353) reported that they received the kits from a peer-leader who belongs to the same social network that they belonged to. When asked how many HIV self-test kits that they received from their peer-leaders, 79.7% (n=283) reported that they received two HIV self-test kits; 19.7% (n=70) received only one kit while 0.6% (n=2) reported that they received three or more kits. Men in Buvuma were significantly less likely to receive two HIV self-test kits than those in Kalangala (74.6% [n=135] *vs.* 85.1% [n=148], *P*=0.002). Approximately 87.9% (n=312) reported that they felt comfortable or very comfortable to receive HIV self-test kits from the peer-leader who gave them the kits.

Table 2. Receipt of HIV self-test kits from peer-leaders by male fisherfolk in Kalangala and Buvuma districts

**Variable**	**Kalangala (n/N, %)**	**Buvuma (n/N, %)**	**Total (n/N, %)**
**No. of men interviewed at baseline**	**N=189**	**N=211**	**N=400**
Number (%) of men interviewed at follow-up	175 (92.6)	186 (88.1)	361 (90.2)
Number (%) of men interviewed at follow-up who received kits from their peer-leaders	174 (99.4)	181 (97.3)	355 (98.3)
**Peer-leader who gave you the kits belongs to the same social network group that you consider to be your primary social network group**			
	**N=174**	**N=181**	**N=355**
Yes	172 (98.8)	181(100)	353 (99.4)
No	2 (1.1)	0 (0.0)	2 (0.6)
**Number of kits that men received from their peer-leaders**			
	**N=174**	**N=181**	**N=355**
1 kit	25 (14.4)	45 (24.9)	70 (19.7)
2 kits	148 (85.1)	135 (74.6)	283 (79.7)
3+ kits	1 (0.6)	1 (0.5)	2 (0.1)
**How comfortable was it for you to receive HIV self-test kits from this person?**			
	**N=174**	**N=181**	**N=355**
Comfortable	104 (59.8)	71 (39.2)	175 (49.3)
Very comfortable	49 (28.2)	88 (48.6)	137 (38.6)
Uncomfortable	10 (5.7)	7 (3.9)	17 (4.8)
Very uncomfortable	11 (6.3)	13 (7.8)	24 (6.8)
Not sure	0 (0.0)	2 (1.1)	2 (0.6)

Table 3 shows the number and proportion of men that distributed the second kit to someone else. There were 283 men who reported that they received two HIV self-test kits from their peer-leaders. Nearly all men (95.0%, n=269) reported that they think it would be a good thing for men to take HIV self-test kits to their female sexual partners. When asked if they gave out the second kit to anyone, 64.0% [illegible]=181) responded in the affirmative, with more men in Buvuma (69.6%, n=94) reporting that they gave out the second kit to anyone than men in Kalangala (58.8%, n=87). Men reported that approximately 97.8% (n=177) of those that they gave the kits to actually accepted to take them. When asked about how easy or difficult it was for them to give out the second kit to those that they gave the kits to, 98.6% (n=143) reported that it was easy or very easy to do so.

Table 3. Distribution of the second kit by male fisherfolk to other people in the fishing communities in Kalangala and Buvuma districts

**Variable**	**Kalangala (n/N, %)**	**Buvuma (n/N, %)**	**Total (n/N, %)**
**Do you think it would be a good thing for men to take HIV self-test kits to their female sexual partners?**			
	**N=148**	**N=135**	**N=283**
Yes	136 (91.9)	133 (98.5)	269 (95.0)
No	6 (4.0)	2 (1.5)	8 (3.0)
Don’t know/not sure	6 (4.0)	1 (0.7)	7 (2.0)
**Did you give out the second kit to anyone to use for HIV self-testing?**			
	**N=148**	**N=135**	**N=283**
Yes	87 (58.8)	94 (69.6)	181 (64.0)
No	61 (41.2)	41 (30.4)	102 (36.0)
**Did the person that you gave the second kit accept to take it?**			
	**N=87**	**N=94**	**N=181**
Yes	85 (97.7)	92 (97.9)	177 (97.8)
No	2 (2.3)	2 (2.1)	4 (2.2)
**Did you find it easy or difficult to give out the second kit to whoever you gave it to?**			
	**N=60**	**N=85**	**N=145**
Very easy	38 (63.3)	61 (71.8)	99 (68.3)
Easy	21 (35.0)	23 (27.2)	44 (30.3)
Difficult	1 (1.7)	1 (1.2)	2 (1.4)
Very difficult	0 (0.0)	0 (0.0)	0 (0.0)

Table 4 shows HIV self-testing uptake and linkage to HIV care among female sexual partners of the male fisherfolk. Of the 177 people who accepted to take kits, 74.6% (n=132) were female sexual partners of the male fisherfolk. Eighty-seven per cent of the male fisherfolk (n=115) reported that their female sexual partners actually used the kits to self-test for HIV. When asked if they tested together with their partners, 73.9% (n=85) of the men responded in the affirmative, with a higher proportion of men in Buvuma than Kalangala (78.3%, n=54 *vs.* 67.4%, n=31; *P*=0.193) reporting that they self-tested together with their female partners. Ninety-seven per cent of the men (n=112) reported that they knew their female sexual partners' HIV self-test results (either through being told by them or through couples' HIV self-testing). Of these, 93.7% (n=105) reported that their female sexual partners were HIV-negative while 6.3% (n=7) were HIV-positive. Only 28.6% (n=2) of the female sexual partners with HIV-positive self-test results were reported to have linked to HIV care.

Table 4. HIV self-testing uptake and linkage to HIV care among female sexual partners of male fisherfolk, as reported by the men

**Variable**	**Kalangala (n/N, %)**	**Buvuma (n/N, %)**	**Total (n/N, %)**
**What was your relationship with the person that you gave the second kit to?**			
	**N=85**	**N=92**	**N=177**
Primary/steady partner	31 (36.5)	59 (64.1)	90 (50.8)
Casual partner	2 (2.3)	3 (3.3)	5 (2.8)
Girlfriend	26 (30.6)	11 (12.0)	37 (20.9)
Relative	4 (4.7)	4 (4.3)	8 (4.5)
Other person	22 (25.9)	15 (16.3)	37 (20.9)
**Proportion of male fisherfolk reporting that their female sexual partners (*primary/steady partner/casual partner/girlfriend*) used/did not use the kits**			
	**N=59**	**N=73**	**N=132**
Used kit	46 (77.9)	69 (94.5)	115 (87.1)
Did not use the kit	8 (13.6)	2 (2.7)	10 (7.6)
Don't know	5 (8.5)	2 (2.7)	7 (5.3)
**Proportion of men reporting that they self-tested together with their female sexual partners, among those that used the kit**			
	**N=46**	**N=69**	**N=115**
Yes	31 (67.4)	54 (78.3)	85 (73.9)

No	15 (32.6)	15 (21.7)	30 (26.1)
**Proportion of men reporting that they knew their female sexual partners' HIV self-test results (*either through female partner telling them or testing together with them*)**			
	**N=46**	**N=69**	**N=115**
Yes	46 (100)	66 (95.6)	112 (97.4)
No	0 (0.0)	3 (4.3)	3 (2.6)
**HIV self-test results of female sexual partners as reported by male fisherfolk**			
	**N=46**	**N=66**	**N=112**
HIV negative	45 (97.8)	60 (90.9)	105 (93.7)
HIV positive	1 (2.2)	6 (9.1)	7 (6.3)
**Linkage to HIV care among HIV-positive female sexual partners as reported by male fisherfolk**			
	**N=1**	**N=6**	**N=7**
Yes	0 (0.0)	2 (33.3)	2 (28.6)
No	1 (100)	3 (50.0)	4 (57.1)
Don't know	0 (0.0)	1 (16.7)	1 (14.3)

Discussion

Our study assessed the acceptability of secondary distribution of HIV self-test kits from the male fisherfolk to their female sexual partners in a fishing community setting. Study findings show that: a) 64% of the men who received two kits gave out the second kit to someone else, of these, 75% gave them to their female sexual partners; b) 87% of female sexual partners who received the kit from the male fisherfolk used them to self-test for HIV (74% together with their male partners), but c) only 29% of HIV-positive female self-testers were reported to have linked to HIV care. In general, these findings show that distribution of HIV self-test kits from the male fisherfolk to their female sexual partners is acceptable with a high proportion of HIV self-testing uptake but linkage to HIV care remains sub-optimal, requiring innovative approaches that are probably aligned to the women's way of life. Some reports indicate that women in the fishing communities are highly mobile [25, 31]; this is likely to have been the reason why those who were HIV-positive failed to

link to HIV care. Thus, any innovative approaches intended to link these women to HIV care should consider their high mobility patterns coupled with the general fisherfolk's reluctance to utilize health facility-based services due to their location far away from the fishing sites [30, 31].

Our finding that only 64% of men that received two kits gave out the second kit to someone else suggests that some male fisherfolk, especially in Kalangala (41.2%) than in Buvuma (30.4%), opted to either keep the second kit for their own use or sold it off for money. However, the finding that nearly all people who were given the second kit (98%) accepted to take them is a clear indication of the potential demand for kits, which could be met with increased distribution through alternative HIV self-test kits distribution channels. It is encouraging that nearly three-quarters of the male fisherfolk gave the kits to their female sexual partners, and that 87% of the female sexual partners who received the kits actually used them to self-test for HIV. Besides, 95% of the male fisherfolk reported that it would be a good thing for men to take HIV self-test kits to their female sexual partners. These findings suggest that secondary distribution of HIV self-test kits from males to their female sexual partners is feasible and can increase HIV testing among female sexual partners of male fisherfolk in a setting characterized by high mobility patterns among women [25]. Also, although we did not primarily aim to assess the uptake of couples' HIV self-testing as part of the main study, our findings that 74% of the women who self-tested for HIV actually tested together with their male partners suggest that the delivery of HIV self-test kits by men to their female partners could have motivated men to self-test together with their female sexual partners. Thus, secondary distribution of HIV self-test kits by males to their female sexual partners can have additional benefits beyond female partner HIV testing, including couples' HIV testing, given that in most cases women are interested in testing together with their male partners but men tend to be

reluctant to do so [22]. Further research is warranted to assess the potential of male partner-delivered HIV self-test kits in improving couples' HIV testing in established relationships within the fishing communities.

We found that linkage to HIV care among the HIV-positive females was low, with only 29% of HIV-positive women being linked to HIV care. If we assume that all the seven women who self-tested HIV-positive were first-time HIV-positive testers, then, this would suggest that up to 71% of women who tested HIV-positive after receiving HIV self-test kits from their male partners did not link to HIV care. As noted above, women may have faced barriers to linkage to HIV care that are related to their high mobility patterns coupled with residence in remote fishing locations that are far away from the main health facilities [25, 30, 31]. Efforts to improve linkage to HIV care among HIV-positive women living in the fishing communities will require innovative approaches that are suitable to women's mobility patterns. These approaches, including use of community health outreaches to the fishing communities, can help to improve linkage to HIV care among highly mobile HIV-positive women [32, 33]. Evidence suggests that community-based ART initiation can help to overcome some of the barriers that unreachable populations, such as those living in the fishing communities, often face in linking to HIV care [34]. However, given that current evidence is based on studies conducted outside fishing community settings, further research is needed to understand if community-based health outreaches can help to improve linkage to HIV care among highly mobile HIV-positive women in the fishing communities.

This study had a number of limitations and strengths. First and foremost, the study relied on self-reports about the female partners' use of HIV self-test kits to test for HIV. We don't know if women

performed the self-test or interpreted their results correctly, although our assumption is that they were trained on how to use the kits and interpret their results by their male partners who had used the kits previously. But, even then, we don't know if these women were first-time HIV-positive testers, and if so, whether or not they sought confirmatory HIV testing, as recommended. Thus, our study findings, based on men's reports of their female sexual partners' HIV self-testing behaviors, should be interpreted with caution. Future studies may aim to interview the female partners themselves, to not only document if they used the kits and interpreted their HIV self-test results correctly but also to document if they were first-time HIV-positive testers, if they received HIV-positive self-test results. In this way, it would be possible to document if the first-time, HIV-positive testers sought confirmatory HIV testing and/or linked to HIV care, depending on the outcome of the confirmatory test. Nevertheless, despite these limitations, our study explored a novel approach for reaching women in the fishing communities with HIV self-testing through their male partners. Our findings indicate a promising HIV self-test kits distribution approach, and, if adopted across settings, secondary distribution of HIV self-test kits from men to their female partners could be the game changer needed to improve HIV testing uptake among highly mobile women in the fishing communities who tend to test less frequently than their male counterparts [35].

Conclusion

Our findings show that secondary distribution of HIV self-test kits from male fisherfolk to their female sexual partners is acceptable in a fishing community setting but linkage to HIV care among self-tested HIV-positive women remained sub-optimal. These findings suggest a need for innovative approaches to improve linkage to HIV care among HIV-positive women in the fishing

communities who are usually mobile and may face challenges accessing conventional HIV services due to their high mobility patterns.

Acknowledgements

We acknowledge the support of the district and community leadership in supporting the implementation of the study; the peer-leaders in Kalangala and Buvuma for distributing the HIV self-test kits to men within their social networks; research assistants for collecting the data, and the study participants for their time in participating in the study.

References

1. Mweemba O, Maman S. The secondary distribution of HIV self-testing kits. Lancet Glob Health. 2021; 9(7):e891-e892.
2. Naughton B, Bulterys MA, Mugisha J, Mujugira A, Boyer J, Celum C, et al. 'If there is joy… I think it can work well': a qualitative study investigating relationship factors impacting HIV self-testing acceptability among pregnant women and male partners in Uganda. BMJ Open. 2023; 13(2):e067172.
3. Joseph Davey DL, Wall KM, Naidoo N, Naidoo D, Xaba G, Serao C, et al. HIV testing and linkage to ART following secondary distribution of HIV self-test kits to male partners of women living with HIV: a pilot randomized control trial in Mpumalanga, South Africa. J Int AIDS Soc. 2022; 25(6):e25937.
4. Ky-Zerbo O, Desclaux A, Boye S, Vautier A, Rouveau N, Kouadio BA, et al. Willingness to use and distribute HIV self-test kits to clients and partners: A qualitative analysis of female sex workers' collective opinion and attitude in Côte d'Ivoire, Mali, and Senegal. Womens Health (Lond). 2022; 18: 17455057221092268.
5. Thirumurthy H, Masters SH, Mavedzenge SN, Maman S, Omanga E, Agot K. Promoting male partner HIV testing and safer sexual decision making through secondary distribution of self-tests by HIV-negative female sex workers and women receiving antenatal and post-partum care in Kenya: a cohort study. Lancet HIV. 2016;3(6):e266-74.
6. Masters SH, Agot K, Obonyo B, Napierala Mavedzenge S, Maman S, Thirumurthy H. Promoting Partner Testing and Couples Testing through Secondary Distribution of HIV Self-Tests: A Randomized Clinical Trial. PLoS Med. 2016;13(11):e1002166.
7. Korte JE, Kisa R, Vrana-Diaz CJ, Malek AM, Buregyeya E, Matovu JKB, et al. HIV Oral Self-Testing for Male Partners of Women Attending Antenatal Care in Central Uganda: Uptake of Testing and Linkage to Care in a Randomized Trial. J Acquir Immune Defic Syndr. 2020; 84(3):271-279.
8. Gichangi A, Wambua J, Mutwiwa S, Njogu R, Bazant E, Wamicwe J, et al. Impact of HIV Self-Test Distribution to Male Partners of ANC Clients: Results of a Randomized Controlled Trial in Kenya. J Acquir Immune Defic Syndr. 2018; 79(4):467-473.

9. Marwa T, Karanja S, Osero J, Orago A. The effects of HIV self-testing kits in increasing uptake of male partner testing among pregnant women attending antenatal clinics in Kenya: a randomized controlled trial. Pan Afr Med J. 2019; 33:213.
10. Maman S, Murray KR, Napierala Mavedzenge S, Oluoch L, Sijenje F, Agot K, et al. A qualitative study of secondary distribution of HIV self-test kits by female sex workers in Kenya. PLoS One. 2017; 12(3):e0174629.
11. Matovu JKB, Kisa R, Buregyeya E, Chemusto H, Mugerwa S, Musoke W, et al. 'If I had not taken it [HIVST kit] home, my husband would not have come to the facility to test for HIV': HIV self-testing perceptions, delivery strategies, and post-test experiences among pregnant women and their male partners in Central Uganda. Glob Health Action. 2018; 11(1):1503784.
12. Ware NC, Wyatt MA, Pisarski EE, Kamusiime B, Kasiita V, Nalukwago G, et al. How pregnant women living with HIV and their male partners manage men's HIV self-testing: qualitative analysis of an HIVST secondary distribution process in Kampala, Uganda. J Int AIDS Soc. 2023; 26(1):e26050.
13. Matovu JKB, Mbita G, Hamilton A, Mhando F, Sims WM, Thompson N, et al. Men's comfort in distributing or receiving HIV self-test kits from close male social network members in Dar Es Salaam, Tanzania: baseline results from the STEP project. BMC Public Health. 2021; 21(1):1739.
14. Sithole N, Koole O, Sausi K, Krows M, Schaafsma T, et al. Secondary Distribution of HIV Self-Testing Kits to Social and Sexual Networks of people living with HIV in KwaZulu-Natal, South Africa. A Brief Report. Front Public Health. 2022; 10:855625.
15. Lightfoot MA, Campbell CK, Moss N, Treves-Kagan S, Agnew E, Kang Dufour MS, et al. Using a Social Network Strategy to Distribute HIV Self-Test Kits to African American and Latino MSM. J Acquir Immune Defic Syndr. 2018;79(1):38-45.
16. Lippman SA, Lane T, Rabede O, Gilmore H, Chen YH, Mlotshwa N, et al. High Acceptability and Increased HIV-Testing Frequency after Introduction of HIV Self-Testing and Network Distribution Among South African MSM. J Acquir Immune Defic Syndr. 2018; 77(3):279-287.
17. Lyu H, Feng Y, Zhou Y, Guo Z, Wang Y, Cui M, et al. Factors Associated with First-Time HIV Testing Among MSM via Secondary Distribution of HIV Self-test Kits in Zhuhai, China. AIDS Behav. 2023; 27(6):1942-1949.

18. Sougou NM, Bassoum O, Faye A, Leye MMM. Women's autonomy in health decision-making and its effect on access to family planning services in Senegal in 2017: a propensity score analysis. BMC Public Health. 2020; 20(1):872.
19. Andriano L, Behrman J, Monden C. Husbands' Dominance in Decision-Making About Women's Health: A Spatial Diffusion Perspective in Sub-Saharan Africa. Demography. 2021; 58(5):1955-1975.
20. Alemayehu M, Meskele M. Health care decision making autonomy of women from rural districts of Southern Ethiopia: a community based cross-sectional study. Int J Womens Health. 2017; 9:213-221
21. Nepal A, Dangol SK, Karki S, Shrestha N. Factors that determine women's autonomy to make decisions about sexual and reproductive health and rights in Nepal: A cross-sectional study. PLOS Glob Public Health. 2023; 3(1):e0000832.
22. Matovu JK, Wanyenze RK, Wabwire-Mangen F, Nakubulwa R, Sekamwa R, Masika A, et al. "Men are always scared to test with their partners … it is like taking them to the Police": Motivations for and barriers to couples' HIV counselling and testing in Rakai, Uganda: a qualitative study. J Int AIDS Soc. 2014; 17(1):19160.
23. Petros G, Airhihenbuwa CO, Simbayi L, Ramlagan S, Brown B. HIV/AIDS 'othering' in South Africa: the blame goes on. Cult Health Sex. 2006; 8(1):67-77
24. Lekalakala-Mokgele E. Exploring gender perceptions of risk of HIV infection and related behaviour among elderly men and women of Ga-Rankuwa, Gauteng Province, South Africa. SAHARA J. 2016; 13(1):88-95.
25. Kwena, Z, Nakamanya S, Nanyonjo G. *et al.* Understanding mobility and sexual risk behaviour among women in fishing communities of Lake Victoria in East Africa: a qualitative study. BMC Public Health. 2020; 20:944.
26. Bahemuka UM, Okimat P, Webb EL, Seeley J, Ssetaala A, Okech B, et al. Factors Associated with Short and Long Term Mobility and HIV Risk of Women Living in Fishing Communities Around Lake Victoria in Kenya, Tanzania, and Uganda: A Cross Sectional Survey. AIDS Behav. 2023; 27(3):880-90.
27. Parliament of Uganda. Report of the Committee on HIV/AIDS on its oversight visits to fish landing sites. Kampala: Parliament of Uganda; March 2015. Available online at: https://www.parliament.go.ug/cmis/browser/Sites/parliament/documentLibrary/Committee%20

Reports/Standing%20Committees/Committee%20on%20HIV-AIDS%20and%20Related%20Matters

28. Agiresaasi A. Region's Remoteness Thwarts Efforts to Eliminate HIV. Available online at: https://globalpressjournal.com/africa/uganda/fishing-community-keeps-catching-hiv/.
29. Ministry of Health. Preliminary results of the 2020 Uganda Population-based HIV Impact Assessment (UPHIA). Kampala: Ministry of Health. Available online at: https://www.mediacentre.go.ug/sites/default/files/media/MOH%20Statement_0.pdf.
30. Bogart LM, Naigino R, Maistrellis E, Wagner GJ, Musoke W, Mukasa B, et al. Barriers to Linkage to HIV Care in Ugandan Fisherfolk Communities: A Qualitative Analysis. AIDS Behav. 2016; 20(10):2464-2476.
31. Tumwine C, Aggleton P, Bell S. Accessing HIV treatment and care services in fishing communities around Lake Victoria in Uganda: mobility and transport challenges. Afr J AIDS Res. 2019; 18(3):205-214.
32. Toms K, Potter H, Balaba M, Parkes-Ratanshi R. Efficacy of HIV interventions in African fishing communities: A systematic review and qualitative synthesis. Int J Infect Dis. 2020; 101: 326-33.
33. Sanga ES, Lerebo W, Mushi AK, Clowes P, Olomi W, Maboko L, Zarowsky C. Linkage into care among newly diagnosed HIV-positive individuals tested through outreach and facility-based HIV testing models in Mbeya, Tanzania: a prospective mixed-method cohort study. BMJ Open. 2017; 7(4): e013733.
34. Eshun-Wilson I, Awotiwon AA, Germann A, Amankwaa SA, Ford N, Schwartz S, et al. Effects of community-based antiretroviral therapy initiation models on HIV treatment outcomes: A systematic review and meta-analysis. PLoS Med. 2021; 18(5): e1003646.
35. Radmanovic J. Biometric fingerprint technology for estimating frequent HIV testing and HIV incidence among mobile men and women in fishing communities along Lake Victoria, Uganda. Master's Thesis. Gothenburg University Library, Gothenburg University, Gothenburg, Sweden. Available at: https://gupea.ub.gu.se/handle/2077/72716

Supplementary Information

S1. Dataset used in the analysis of data.

S2. STROBE Checklist

Dataset

Click here to access/download
Supporting Information
S1_Dataset_used_in_the_analysis.dta

STROBE Checklist

Click here to access/download
Supporting Information
S2_STROBE_Checklist_PEST4MEN.doc
