# Supplementary material for: Secondary distribution of HIV self-test kits from males to their female sexual partners in two fishing communities in rural Uganda
Source: PLOS Glob Public Health. 2023 Nov 29;3(11):e0002477. doi: 10.1371/journal.pgph.0002477 (PMC10686447; doi:10.1371/journal.pgph.0002477)
Supplement: Attachment — Submitted filename: Response_to_Reviewers_Comments.doc [file pgph.0002477.s004.doc]

**RESPONSE TO EDITORS COMMENTS**

The main objective of this manuscript is to assess the acceptability of Secondary distribution of HIV self-test kits from male fisherfolk to their female sexual partners, however, it is noted that nearly 40% of the men participating in the study are not involved in fishing or fishing related occupations- please clarify or alternatively revise the title and objectives accordingly.

**Response: We have revised the title and the corresponding text in the manuscript to refer to “men in the fishing communities” rather than “male fisherfolk”. The term “men in the fishing communities” is more encompassing and caters for all categories of men that we enrolled into the study. The change has been reflected across the paper, as shown in track changes.**

While you sought to assess the acceptability of secondary distribution of HIV self test kits- the acceptability is self reported by the participants (you have mentioned this in your limitations)- the tests may have been forced on the partners or falsely reported- there is therefore no true evidence of the acceptability of the tests- and, therefore, may be termed 'reported acceptability.'

**Response: We have opted to use the term ‘reported acceptability’, as suggested, given that the recipients of the second kit were not actually interviewed but only the men who distributed the kits to them were interviewed about the use of the kits among those that they gave kits to. The changes have been made across the paper, as shown in track changes.**

How was the sample size of 400 participants decided?

**Response: On page 5, we have indicated how the sample size of 400 men was determined. In summary, each trained peer-leader recommended 20 men from their social network; and, with up to 22 peer-leaders selected and trained, a total of 475 men were screened for study eligibility. It is from this number that a sample of 400 men were selected and enrolled into the primary study. However, since this is a secondary analysis of data from the primary study, we have restricted the information to that which is needed to understand how the sample size of 400 men was determined. This helps us to avoid duplicating the same information in this paper with that in the primary paper that’s still under review. We will await additional guidance on this issue from the Editor if we need to provide additional information in this regard.**

Were the participants taught how to use the test?  Were they told to communicate how to use the testing kit to those they gave it to?- please include this in the methods.

**Response: We have included information indicating that the peer-leaders were trained on how to use the kits and they were requested to sensitize their social network members through a practical demonstration on how the kit is used and how to read and interpret results. See pages 5-6**.

Results- Actual numbers to be mentioned with percentages in brackets.

**Response: We have made this adjustment as suggested, throughout the results section.**

Given that the characteristics of the partners who accepted the tests are not available, predictors of having shared the test or both partners having done the test may be presented.

**Response: We thank the Editor for this suggestion. However, since the participants did not only give kits to their female sexual partners but also to other people, including their relatives, we felt that performing inferential statistics to determine the predictors of sharing the kit with anyone would not yield the predictors associated with giving the kit to a female sexual partner --- yet this is the primary focus of the paper. To address this challenge, we went ahead to categorize those that received the kits into two groups: a) female sexual partners and b) other recipients. We then conducted bivariate and multivariable analyses to determine the probability that the recipient of the second kit was a female sexual partner. As noted in Table 5, men were more likely to give the second kit to a female sexual partner if they were married or had difficulties in reading text prepared in the local language; i.e. *Luganda*.**

Kindly avoid assumptions such as for example: 'kept the second test for their own use or sold it for money' without sufficient evidence.

**Response: We have edited the text to remove any assumptions. We have indicated that some of the actual reasons will be captured as part of the planned post-intervention qualitative assessment.**

Limitations need to include why the women who received the tests were not included. Likely biases such as selection bias (i.e., regarding those who received the kits) need to be discussed. How likely is it that the women willingly accepted the test? While this has been mentioned in the need for the study there is no information regarding this subsequently. The data was secondary- a comment on the quality of the data and how that impacts the results is needed.

**Response: We have expanded the limitation to include a focus on why we did not interview the female partners of the men enrolled into the study. Specifically, as per study design, the primary study was intended to target exclusively men, and the second kit was not tied to anyone – i.e. men were not specifically told to give the second kit to a female sexual partner. It was only at the time of analysis that we realized that ¾ of the men gave the kits to their female sexual partners. So, it is for this reason that no effort was made to interview their female sexual partners. At the moment, we don’t know if the women willingly accepted, among other issues, considering that the reports were obtained from the men rather than from their female sexual partners. These aspects have been reflected in the revised limitations section in the revised paper.**

Please submit your revised manuscript by Oct 01 2023 11:59PM. If you will need more time than this to complete your revisions, please reply to this message or contact the journal office at [globalpubhealth@plos.org](mailto:globalpubhealth@plos.org). When you're ready to submit your revision, log on to <https://www.editorialmanager.com/pgph/> and select the 'Submissions Needing Revision' folder to locate your manuscript file.

Please include the following items when submitting your revised manuscript:

- A rebuttal letter that responds to each point raised by the editor and reviewer(s). You should upload this letter as a separate file labeled 'Response to Reviewers'.
- A marked-up copy of your manuscript that highlights changes made to the original version. You should upload this as a separate file labeled 'Revised Manuscript with Track Changes'.
- An unmarked version of your revised paper without tracked changes. You should upload this as a separate file labeled 'Manuscript'.

Guidelines for resubmitting your figure files are available below the reviewer comments at the end of this letter.

**Journal Requirements**:

1. Please ensure that Funding Information and Financial Disclosure Statement are matched.

2. In the Funding Information you indicated that no funding was received. Please revise the Funding Information field to reflect funding received.

Please use the space provided to explain your answers to the questions above. You may also include additional comments for the author, including concerns about dual publication, research ethics, or publication ethics. (Please upload your review as an attachment if it exceeds 20,000 characters)

Reviewer #1: Firstly, I would like to appreciate the immense effort taken by the authors to carry out this study among a vulnerable population, based on occupation as well as gender. This is an all in all well written paper, although I do have the following reservations about it.

1) a flow chart describing the recruitment and follow-up process will be appreciated. details of number of participants recruited, found eligible, reasons and proportions of ineligibility, lost to follow-up and further outcomes.

**Response: As requested, we have provided additional information regarding the recruitment process in the revised paper (see page 5). However, while we agree with reviewer that a flow chart would be helpful, we have not included it in the revised paper as requested since it would duplicate the same flow chart that is already included in the primary paper. With all due respect, we would like to request that we be allowed to restrict the information to only that which can help the reader to know how the recruitment process was done (like we have done on page 5) but leave out other additional details that already appear in the primary paper. The primary paper is about the feasibility, acceptability and preliminary effects of the intervention on HIV testing uptake, linkage to and retention in HIV care among men, and is thus different from this paper. However, it includes information on how the primary study (from which the data for the secondary analysis have been drawn) was implemented, part of which is the flow chart referred to in this comment. We will be glad to receive additional guidance on this issue from the Editor.**

2) language in which the interview was carried out can be added in the description of data collection

**Response: We have added the language of interview. See page six for details.**

3) how was interviewer bias addressed?

**Response: Interviewer bias was addressed in many ways: a) as noted in the paper, all data collectors were trained on the purpose and objectives of the study to avoid enrolling people who did not meet the eligibility requirements; b) the participants were enrolled from people who had been recommended to the study team by their respective peer-leaders; c) a screening tool with clear instructions was used to select those that were eligible for the study, and d) peer-leaders were involved in checking the authenticity of the participants if doubts arose during the screening, enrolment or follow-up process.**

4) Of those who did not distribute the self test kit, what were the reasons?

**Response: Whatever reasons we have at the moment are probable, and we don’t want to use them, as requested by the Editor as well as reviewer 2. The true reasons will be deciphered as part of the planned post-intervention qualitative assessment – at the end of the study.**

5) A multivariate logistic regression maybe to tease out those variables that have an impact on your primary outcome (as very few did not take up testing kits, one kit v/s 2 or more kits can be the outcome variable).

**Response: We have included a multivariable analysis of the factors associated with giving the second kit to a female sexual partner, in response to the comment from the Editor. Yes, we focused on those who received two kits, and determined those who gave out the second kit to anyone else, including their sexual partners. Of these, we determined the probability that the recipient was female. See Table 5 for details.**

6) Scope for mixed methods studies with the women folk who received the test kits can be mentioned in the limitations of the study

**Response: We have included, as a limitation, the fact that information about the women referred to was obtained from the men rather than from the women themselves. We have also provided reasons why this was the case, and proposed a need for future studies to interview the women themselves rather than rely on reports of HIV self-test kits’ use from their male partners.**

Reviewer #2: The paper requires minor correction. Otherwise, the paper meets the PLOS Global Public Health standards and guidelines.

Line 40: 88.0% of 283 men is not 181.

**Response: The percentage has been corrected. It now reads as 64%.**

Line 110: Why was this an eligible criterion: "Should not have tested for HIV at least three months from the time of enrolment"

**Response: We have now provided reasons for the 3-month cut-off – which aligns with the Ministry of Health’s recommendation for repeat HIV testing after every three months, among key and priority populations of which people living in the fishing communities are part of. See lines 140-144, page** 5.

Line 125: Why was no attmept made to interview the female partners who received the second kit (Because the authors did not attempt to interview the female partners who received the second kit, the second part of the definition of acceptance may remain unfulfilled)

**Response: As mentioned in the discussion section, by design, women were not part of the study. Thus, no attempt was made to reach them because of this reason. However, we acknowledge the limitations of obtaining information about the use of the kits by women from their male sexual partners, and this is well catered for in the ‘Discussion’ section. See pages 18-19.**

Line 158: Why weren't all 400 men engaged in fishing or fishing related activities? More than 40% of the study subjects were involved in non-fishing activities. In that case, the findings of this study cannot be generalized to fisher folk.

**Response: We agree that the findings are not generalizable to the male fisherfolk – since not every man enrolled in the study was a male fisherfolk. Besides acknowledging this limitation, we have revised the study title to change from ‘male fisherfolk’ to ‘men in the fishing communities’. This change has been reflected elsewhere in the manuscript.**

Line 232, 233: Many of the statements in the discussion are 'assumptions' made without any corroboration from the findings of this study. There are some statements that are assumptions, but they are backed by reference. But many aren't. Discussion may have to be re-written.

**Response: We have edited the ‘Discussion’ section to remove any statements based on assumptions; especially those where we don’t have any evidence from our study or from prior studies. However, since not every statement in the discussion section was an assumption or based on assumptions, we have, with due respect, not re-written the discussion section as advised. Instead, we have retained those statements that are well backed up by available evidence but dropped those statements that appear as assumptions without any evidence to back them up. We hope this helps to meet the reviewer’s expectations on this comment.**
